# Lipid droplets in stress protection: distinct mechanisms of lipid droplet microautophagy

**DOI:** 10.1080/27694127.2022.2067643

**Published:** 2022-05-03

**Authors:** Pin-Chao Liao, Liza A. Pon

**Affiliations:** a Department of Pathology and Cell Biology, Columbia University, New York, NY 10032, United States; b Institute of Molecular Medicine, National Tsing Hua University, Hsinchu 30013, Taiwan; c Department of Life Science, National Tsing Hua University, Hsinchu 30013, Taiwan

## Abstract

Lipid droplets (LDs) are organelles that function as sites for lipid storage. LDs have also been implicated in the cellular response to proteotoxic or lipotoxic stress as sites for sequestering dysfunctional or excess proteins or lipids, and targeting those cargos for degradation by LD microautophagy (microlipophagy, µLP). Here, we describe two mechanisms for µLP in yeast, which are triggered by different stressors. µLP occurs at raft-like liquid ordered microdomains in the vacuolar membrane in yeast exposed to severe nutrient limitations. In contrast, in yeast exposed to ER stress or less severe nutrient limitations, LD uptake at the vacuole is liquid ordered (L_o_) microdomain-independent and dependent upon vacuolar membrane remodeling mediated by endosomal sorting complexes required for transport (ESCRT).

Lipid droplets (LDs) are organelles that form in the ER and consist of a phospholipid monolayer surrounding a neutral lipid-rich core. LDs have an established function in lipid homeostasis as a storage site for lipids, which can be mobilized for energy production, membrane expansion and remodeling, and generation of lipid-based signaling molecules and hydrophobic vitamins. Recent studies revealed a role for LDs in cellular stress responses. Indeed, LDs are upregulated by stressors including lipid imbalance, nutrient limitation, starvation and ER stress (accumulation of unfolded proteins in the ER). LDs contribute to protection against proteotoxic or lipotoxic stress by serving as a site for sequestering unfolded proteins or lipids that are damaged or present in excess and targeting those cargos for degradation by LD autophagy.

Microautophagy is a conserved, but poorly understood form of autophagy. During LD microautophagy, known as microlipophagy (µLP), LDs contact the lysosome (the vacuole in yeast), and partial or wholesale uptake of LDs into the lysosome/vacuole occurs at sites of invagination in the lysosome/vacuole membrane. µLP has emerged as the primary mechanism for stressor-induced degradation of LDs and their toxic cargos in the budding yeast, *Saccharomyces cerevisiae*. Here, we describe two mechanisms for µLP in yeast, which are triggered by different stressors, occur at distinct domains in the vacuolar membrane and utilize different mediators for vacuolar membrane invagination and scission [1].

One mechanism for stressor-induced µLP occurs at liquid ordered (L_o_) microdomains, which are lipid raft-like regions that are sterol-rich and have distinct protein and lipid composition compared to the bulk of the vacuolar membrane. Vph1, a component of the vacuolar-type H^+^-translocating ATPase, is excluded from L_o_ microdomains. In contrast, sterol transporters (Lam6/Ltc1 and Nce102), as well as target of rapamycin complex 1 (TORC1) subunits (Tco89) and modulators (Ivy1, Gtr1, Gtr2 and Iml1) are enriched in L_o_ microdomains. Interestingly, L_o_ microdomain formation is induced by all stressors studied. However, the mechanisms for L_o_ microdomain formation and function in µLP are stressor-specific.

L_o_ microdomains mediate µLP in response to severe nutrient limitations that block cell proliferation (entry into stationary phase, nitrogen starvation or lipid imbalance). Specifically, L_o_ microdomains have been implicated as sites for interaction of LDs with vacuoles and invagination of the vacuolar membrane during µLP in stationary-phase or nitrogen-starved yeast. Moreover, inhibition of L_o_ microdomain formation inhibits LD uptake in stationary-phase or nitrogen-starved yeast and partially inhibits µLP in yeast exposed to lipid imbalance. Interestingly, the L_o_ microdomain-associated protein Ivy1 contains a putative I-BAR domain, which binds to and stabilizes membranes with negative membrane curvature. Ivy1 can also bind to Ypt7, the RAB7 GTPase of yeast, and requires Ypt7 for localization to invaginations in the vacuolar membrane in response to nutrient limitation. Thus, Ivy1 may mediate invagination of the vacuolar membrane at L_o_ microdomains during µLP-associated uptake of LDs into the yeast vacuole.

On the other hand, µLP in yeast exposed to ER stress or mild glucose limitation that triggers a shift from glycolysis- to respiration-driven growth (the diauxic shift) does not require L_o_ microdomains. Instead, this form of µLP depends on the endosome sorting complex required for transport (ESCRT). ESCRT functions in membrane invagination and scission during processes including multivesicular body (MVB) formation, the abscission phase of cytokinesis and budding of viruses from the plasma membrane. Although MVBs can contribute to L_o_ microdomain formation by delivering sterols to the vacuole in nitrogen-starved cells, deletion of the ESCRT-III component *SNF7* does not affect L_o_ microdomain formation under ER stress conditions. In contrast, deletion of ESCRT components blocks µLP in cells exposed to ER stress and partially inhibits µLP in response to lipid imbalance, a condition that induces ER stress. Moreover, ER stress induces recruitment of ESCRT components to the vacuolar membrane and to sites of vacuolar membrane invagination during LD uptake into the organelle during ER stress. Collectively, these data indicate the direct role of ESCRT in µLP through its function in vacuolar membrane remodeling and scission.

In summary, µLP functions in mitigation of cellular stress in yeast by removal of LDs and their associated stress-induced, toxic cargos. µLP relies on formation of lipid raft-like L_o_ microdomains in cells exposed to nutrient limitations that are severe and block cell proliferation. However, in yeast exposed to ER stress and less severe nutrient limitations, LD uptake at the vacuole is L_o_ microdomain-independent and dependent upon vacuolar membrane remodeling mediated by ESCRT (Fig. 1). Because LD biogenesis and µLP are stress responses in mammalian cells, it is possible that the mechanisms identified in yeast may also be conserved. Finally, it is not clear why there are two distinct mechanisms for LD uptake at the vacuolar membrane in yeast. Because L_o_ microdomains are less abundant and mature in stressed mid-log phase cells compared to stationary phase cells, it is possible that the µLP mechanism used for stress control is linked to L_o_ microdomain formation.

**Figure 1.**
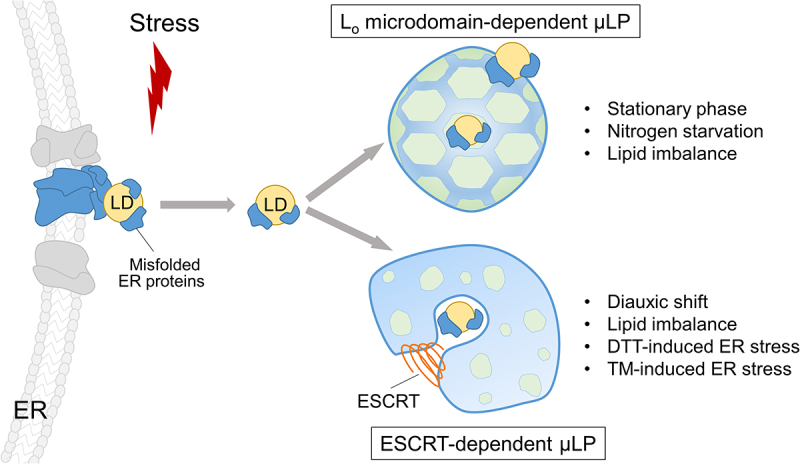
LD-mediated ER proteostasis. L_o_ microdomains are formed in response to various stressors. LDs remove misfolded proteins from the ER and are directly taken up into vacuoles (yeast lysosome analogs) for degradation by L_o_ microdomain-dependent or/and ESCRT-dependent µLP. Stressors that induce different types of µLP are shown. This novel ER protein quality control pathway provides an alternative mechanism to remove misfolded proteins from the ER in bulk.
